# Reviewers

**DOI:** 10.1111/jdi.12292

**Published:** 2014-10-27

The publication of invaluable papers in the *Journal of Diabetes Investigation* depends on the prompt, careful review of submitted manuscripts. We would like to thank the Editorial Board members, the International Editorial Board members, the Assistant Editorial Board members and the following experts for reviewing manuscripts from October 1, 2013 to August 31, 2014.

Hideharu Abe

Toru Aizawa

Keiko Arai

Atsushi Araki

Shin-ichi Araki

Yoshimasa Aso

Abdul Basit

Phillip Brantley

Dehong Cai

Lu Cai

Juliana Chan

Fang-Rong Chang

Sang Ah Chang

Tien-Jyun Chang

Harn-Shen Chen

Liming Chen

Ken Chiu

Dong Hyeok Cho

Jae Hyoung Cho

Yong Jun Choi

Shao-Yuan Chuang

Daisuke Chujo

Sung Wan Chun

Makoto Daimon

Sam J Daniel

Takahisa Deguchi

Toshio Doi

Nabil El-Naggar

Masanori Emoto

Kei Fujimoto

Shimpei Fujimoto

Tomomi Fujisawa

Yukihiro Fujita

Michiaki Fukui

Mitsuo Fukushima

Kengo Furuichi

Hiroto Furuta

Carlo Galli

Kathleen M Gillespie

Atsushi Goto

Honglei Guo

Yusuke Hada

Akihiro Hamasaki

Naotake Hashimoto

Mitsuru Hashiramoto

Tomoshige Hayashi

Yasuaki Hayashino

Takumi Hirata

Takahisa Hirose

Yushi Hirota

Timothy Hla

Yen-Ping Ho

Yukio Horikawa

Masaaki Hoshiga

Masayuki Hosoi

Chang-Hsun Hsieh

Kuo-Chin Huang

Cn Huang

Jee-Fu Huang

Yanan Huo

You-Cheol Hwang

Chii-Min Hwu

Md. Auwal Ibrahim

Atsuhiro Ichihara

Kazuo Inoue

Kouichi Inukai

San-e Ishikawa

Masahiro Iwasaki

Masanori Iwase

Lijing Jia

Jyuhn-Huarng Juang

Tetsuya Kakuma

Naomi Kamimura

Hideki Kamiya

Hideaki Kaneto

Naoto Katakami

Tomonori Kato

Koichi Kato

Takumi Kawaguchi

Daiji Kawanami

Toru Kikuchi

Chul-Hee Kim

Chong Hwa Kim

Sang Soo Kim

Jae Hyeon Kim

Jaetaek Kim

Toshihide Kimura

Tetsuro Kobayashi

Daisuke Koya

Hidenori Koyama

Hiroyuki Kubota

Akira Kubota

Shoen Kume

Akio Kuroda

Takeshi Kurose

Johanna Kuusisto

Satoshi Kuwabara

Soo-Heon Kwak

Joseph Kwan

Hyuk-Sang Kwon

A Lacquaniti

Hc Lam

Terence Lao

Byung-Wan Lee

Chien-Hsing Lee

Won-Young Lee

I-Te Lee

Jenny Lee

Ji-Hyun Lee

Yau-Jiunn Lee

Ting-I Lee

Hung-Yuan Li

Qifu Li

Chou-Ching K Lin

Shaoda Lin

Shu-Fu Lin

Ying Lu

Zhaohui Lu

Shiro Maeda

Hiroshi Maegawa

Yuichi Makino

Masafumi Matsuda

Munehide Matsuhisa

Michihiro Matsumoto

Silvia Migliaccio

Tohru Minamino

Jun-ichiro Miyagawa

Satoshi Miyata

Hiroki Mizukami

Tomoaki Morioka

Tatsumi Moriya

Seiichi Munesue

Shoichiro Nagasaka

Tomoko Nakagami

Shuhei Nakanishi

Yasuko Nakano

Takuma Narita

Masami Nemoto

Daniel Ng

Masahiro Nishi

Wataru Nishimura

Yoshihiko Nishio

Mitsuhiko Noda

Yoshinori Noguchi

Makiko Ogata

Jee-Young Oh

Shinichi Oikawa

Etsuji Okamoto

Yoshihisa Okamoto

Nermin Olgun

Christine L Oltman

Hiroshi Onuma

Haruhiko Osawa

Yen-Chuan Ou

Harry Powell

David Rodbard

Yoshifumi Saisho

Kazuhiko Sakaguchi

Naoki Sakane

Hiroshi Sakaue

Kazunori Sango

Hideyuki Sasaki

Masataka Sata

Osamu Sekine

Yi-Jing Sheen

Yunfeng Shen

Tadao Shibasaki

Kenichi Shikata

Hitoshi Shimano

Seiya Shimoda

Kohji Shirai

Hirohito Sone

Noriyuki Sonoda

Kazuhiro Sugimoto

Takashi Sugiyama

Shoichiro Sumi

Zilin Sun

Yuji Tajiri

Mitsuyoshi Takahara

Yoshihiko Takahashi

Iseki Takamoto

Fumihiko Takeuchi

Yasuhiro Takeuchi

Kohzo Takeyabashi

Yoshifumi Tamura

Yasushi Tanaka

Keiji Tanimoto

Hiroto Terashi

Yasuo Terauchi

Haoming Tian

Kazuyuki Tobe

Satoru Tsujii

Daisuke Tsujino

Kohjiro Ueki

Hiroyuki Umegaki

Tatsuhiko Urakami

Takashi Uzu

Jun Wada

Takashi Wada

Hironori Waki

Chih Yuan Wang

Jong Chul Won

Jianzhong Xiao

Kunimasa Yagi

Hiroaki Yagyu

Tomohide Yamada

Yuichiro Yamada

Satoru Yamada

Sho-ichi Yamagishi

Wari Yamamoto

Ritsuko Yamamoto-Honda

Shunsuke Yamane

Tomonori Yamanishi

Shigeo Yamashita

Tao Yang

Hisafumi Yasuda

Hiroshi Yatsuya

Norihide Yokoi

Hirohide Yokokawa

Masayasu Yoneda

Liu Yongjun

Narihito Yoshioka

Ho Yan Ruby Yu

Pei Yu

Xuefeng Yu

Dalong Zhu

